# Pediatric yeast fungaemia species patterns, resistance profiles, and outcomes: a 20-year surveillance study in Paris, France

**DOI:** 10.1016/j.eclinm.2026.104177

**Published:** 2026-09-01

**Authors:** Fanny Alby-Laurent, Thomas Obadia, Maud Gits-Muselli, Marie-Elisabeth Bougnoux, Benoit Brethon, Patricia Mariani, Juliette Guitard, Françoise Botterel, Elisabeth Chachaty, Karine Boukris-Sitbon, the French Mycoses Study Group, Alexandre Alanio, Adela Angoulvant, Nawel Ait-Ammar, Christine Bonnal, Sophie Brun, Naima Dahane, Blandine Denis, Samia Hamane, Najiby Kassis-Chikhany, Maïté Micaelo, André Paugam, Anne-Laure Roux, Olivier Lortholary, Marie Desnos-Ollivier, Fanny Lanternier

**Affiliations:** a Institut Pasteur, Université Paris Cité, National Reference Center for Invasive Mycoses and Antifungals, Mycology Translational Research Group, Mycology Department, Paris, France; b Department of Pediatric Hematology, Immunology et Oncology, Sorbonne University, Armand Trousseau Hospital, APHP, INSERM (CRSA) - UMR_S-938, Paris, France; c Institut Pasteur, Université Paris Cité, Bioinformatics and Biostatistics Hub, Paris, France; d Microbiology Department, Hôpital Robert Debré, APHP, Paris, France; e Université Paris Cité and Université Sorbonne Paris Nord, Inserm, IAME, Paris, F-75018, France; f Mycology Department, Hôpital Necker-Enfants Malades, APHP, Paris, France; g Institut Pasteur, Université Paris Cité, INRAe USC2019, Inserm U1359, Unité Biologie et Pathogénicité Fongiques, Paris, France; h Paediactric Hematology and Immunology Department, Robert Debré Academic Hospital, GHU AP-HP Nord Université Paris Cité, Paris, France; i Service de Parasitologie-Mycologie, Hôpital Saint-Antoine, AP-HP, Centre de Recherche Saint-Antoine, CRSA, Inserm, Sorbonne Université, Paris, France; j Mycology Department, Hôpital Bicêtre, Paris, France; k Microbiology Department, Institut Gustave Roussy, Paris, France; l Infectious Diseases Unit, Hôpital Necker-Enfants Malades, APHP, Paris, France

**Keywords:** Invasive fungal disease, Children, Risk factors, Yeast bloodstream infection, Epidemiology candidemia, Antifungal resistance

## Abstract

**Background:**

The epidemiology of pediatric yeast bloodstream infections remains poorly characterized in high-income settings despite global shifts over the past two decades. As fungal species distribution varies geographically, national surveillance is essential. In France, recent reports have focused predominantly on adults, leaving a significant evidence gap for children.

**Methods:**

We analyzed data from the YEASTS program, a prospective multicentre hospital-based surveillance study conducted in 27 hospitals in the Paris area, France, between Oct 1, 2002, and Dec 31, 2022. All incident episodes of yeast bloodstream infection diagnosed during this period, were eligible. For the primary analysis, we included children and adolescents aged 6 months to 16 years; neonates and recurrent episodes were excluded. Adult cases (>16 years) recorded during the same period were included for comparison. Species identification and antifungal susceptibility testing, using EUCAST standardized method, were performed at the National Reference Center of invasive Mycoses and Antifungals (NRCMA). The primary objective was to describe the epidemiology of yeast bloodstream infections in children and adolescents and compare findings with those observed in adults (>16 years). The main outcomes assessed were species distribution, antifungal susceptibility patterns, treatment strategies, and 30-day all-cause mortality.

**Findings:**

Among 346 pediatric episodes, hematological malignancy was the main underlying condition (20.5%). *Candida albicans* predominated (42%), although non-*albicans Candida* species collectively accounted for a larger proportion of episodes, notably *Candida parapsilosis* (23.5%) and *Candida tropicalis* (10%). Species distribution varied by age: younger children had more *C. parapsilosis*, older more *Pichia kudriavzevii* (syn. *Candida krusei*) and *Nakaseomyces glabratus* (syn. *Candida glabrata*). *Trichosporon asahii* represented 2%. Resistance rates remained low (4% to caspofungin, 8.1% fluconazole) including 4 echinocandin-resistant isolates that harbored S645P mutations in the Fks coding region. 30-day mortality was 13.5%, independently associated with *C. tropicalis*, *N. glabratus*, *T. asahii*, female sex, and ICU admission. Compared with 5889 adult cases, pediatric patients had a distinct species distribution, more frequent prior antifungal exposure, greater use of liposomal amphotericin B, and significantly lower adjusted 30-day mortality.

**Interpretation:**

Pediatric yeast bloodstream infections exhibit distinct epidemiological characteristics, species distribution, antifungal exposure patterns, and outcomes compared with adult infections. Future research should focus on prospective age-stratified studies to better define optimal antifungal treatment strategies, evaluate the clinical significance of emerging and resistant yeast species, and identify factors associated with poor outcomes in pediatric patients.

**Funding:**

Santé Publique France and Institut Pasteur.

**Research in context**

**Evidence before this study**

Yeast bloodstream infections are a leading cause of morbidity and mortality in immunocompromised patients, those with central venous catheters, and individuals undergoing recent surgery. Over the past 15 years, their epidemiology has shifted markedly, driven by the emergence of antifungal resistance and an increasing diversity of causative species. However, most studies have focused on adults or have aggregated neonatal and pediatric data, despite substantial epidemiological differences across age groups. Moreover, because species distribution and resistance patterns vary geographically, robust national data are essential to inform clinical management and public health strategies. We searched PubMed (MEDLINE) from database inception to December 1, 2024 using the terms « Bloodstream yeast infection » OR « Fungemia » OR « Candidemia » AND « Children » OR « Child » OR « Paediatrics » for studies published in English or in French, excluding reviews and case reports. To our knowledge, no study has directly compared the epidemiology of yeast bloodstream infections between adults and children in France or elsewhere in Europe. In Europe, the largest published pediatric cohort was a retrospective multicentre study conducted between 2005 and 2015 across ten countries, reporting 1395 episodes of candidemia, of which 36.4% occurred in neonates; France did not participate in this study. In France, the YEASTS program remains the largest cross-sectional investigation to date, including data of adults and children since 2002. Only adults’ data of this cohort have been already published.

**Added value of this study**

Given all these reasons, we felt it was essential to analyze in detail the epidemiological data on bloodstream yeast infections in children. Between 2002 and 2022, the YEASTS program, which is a prospective, multi-centric, hospital-based surveillance study focusing on patients developing bloodstream yeast infections in 27 centers of Paris area in France, was implemented at the National Reference Center for Invasive Mycoses and Antifungals including polyphasic identification of all isolates, epidemiology, and factors associated with death.

We report 346 episodes in children and compared them with the 5889 episodes in adults from the same surveillance study. Hematological malignancy was the main underlying condition. *Candida albicans* predominated (42%), but non-albicans species collectively exceeded it, notably *Candida parapsilosis* and *Candida tropicalis;* and *Trichosporon asahii* represented 2%. Species distribution varied by age: younger children had more *C. parapsilosis*, while older children had more *Pichia kudriavzevii* (syn. *Candida krusei*) and *Nakaseomyces glabratus* (syn. *Candida glabrata*). Mortality was 13.5%, independently associated with *C. tropicalis*, *N. glabratus*, *T. asahii*, female sex, and Intensive Care Unit stay and higher in children with yeast fungemia resistant to caspofungin, fluconazole or voriconazole. Compared with adults, children had a distinct species distribution, greater prior antifungal exposure, more frequent use of liposomal amphotericin B, and significantly lower adjusted mortality.

**Implications of all the available evidence**

Our findings reveal a distinct epidemiological signature of pediatric yeast bloodstream infections, with hematological malignancy emerging as the predominant underlying condition, in contrast with the surgical predominance reported in adult cohorts and the neonatal intensive care focus of previous pediatric studies. This unique dataset enabled, for the first time within a unified surveillance framework, a direct comparison between pediatric and adult populations, highlighting substantial age-related differences in species distribution, treatment patterns, and outcomes and challenging the extrapolation of adult data to pediatric therapeutic strategies. This study also provides pediatric-specific benchmarks for antifungal resistance in this region, with fluconazole resistance at 8.1% and caspofungin resistance at 4%, supporting the continued efficacy of recommended empirical treatments. These findings highlight the need for pediatric-specific epidemiological data to improve risk stratification and patient management. Future studies should assess whether age-adapted surveillance strategies and antifungal management approaches can further improve prevention, treatment, and outcomes of yeast bloodstream infections in children.

## Introduction

Bloodstream yeast infections are a major cause of morbidity and mortality in immunocompromised patients, patients with central venous catheters or who underwent recent surgery.^1^ The epidemiology of these infections has changed considerably over the last fifteen years, with the emergence of resistances and new species^2^^,^^3^ and differs significantly among pediatric and adult populations.^4^ As this epidemiology could be influenced by the geographical environment, national data are crucial.^5^ However, most of the recent fungemia studies in France focused on adult patients,^6^, ^7^, ^8^, ^9^ leaving a knowledge gap for their counterpart in children in contrast with several other countries.^10^, ^11^, ^12^, ^13^, ^14^, ^15^, ^16^ Due to potential differences between adult and paediactric populations in terms of ecology, pathophysiology, underlying conditions and mortality of bloodstream yeast infections, additional pediatric data is crucial to better identify children at risk of yeast fungemia and improve case management.

A hospital-based surveillance program for patients developing yeast bloodstream infections (YEASTS program) was launched in 2002 in the Paris area, with the participation of 27 university hospital centers (CHU). The aim of this survey was to collect data from a large cohort of patients over two decades, regardless of hospitalization cause, in order to describe the epidemiology of these infections according to time or underlying disease, along with their outcome. In particular, the YEASTS program helped confirm Intensive Care Unit (ICU) hospitalization increased risk of fungemia and provided a better understanding of risk factors in patients with underlying malignancies.^17^^,^^18^ These results however only focused on the adult population.

In this study, we used this database to describe the particular epidemiology of bloodstream yeast infections in children and adolescents (excluding neonates) in Paris hospitals. We further set out to compare our findings to the epidemiology from the adult population, whose data were collected in the meantime.

## Methods

### YEASTS program and study design

**Fig. 1:**
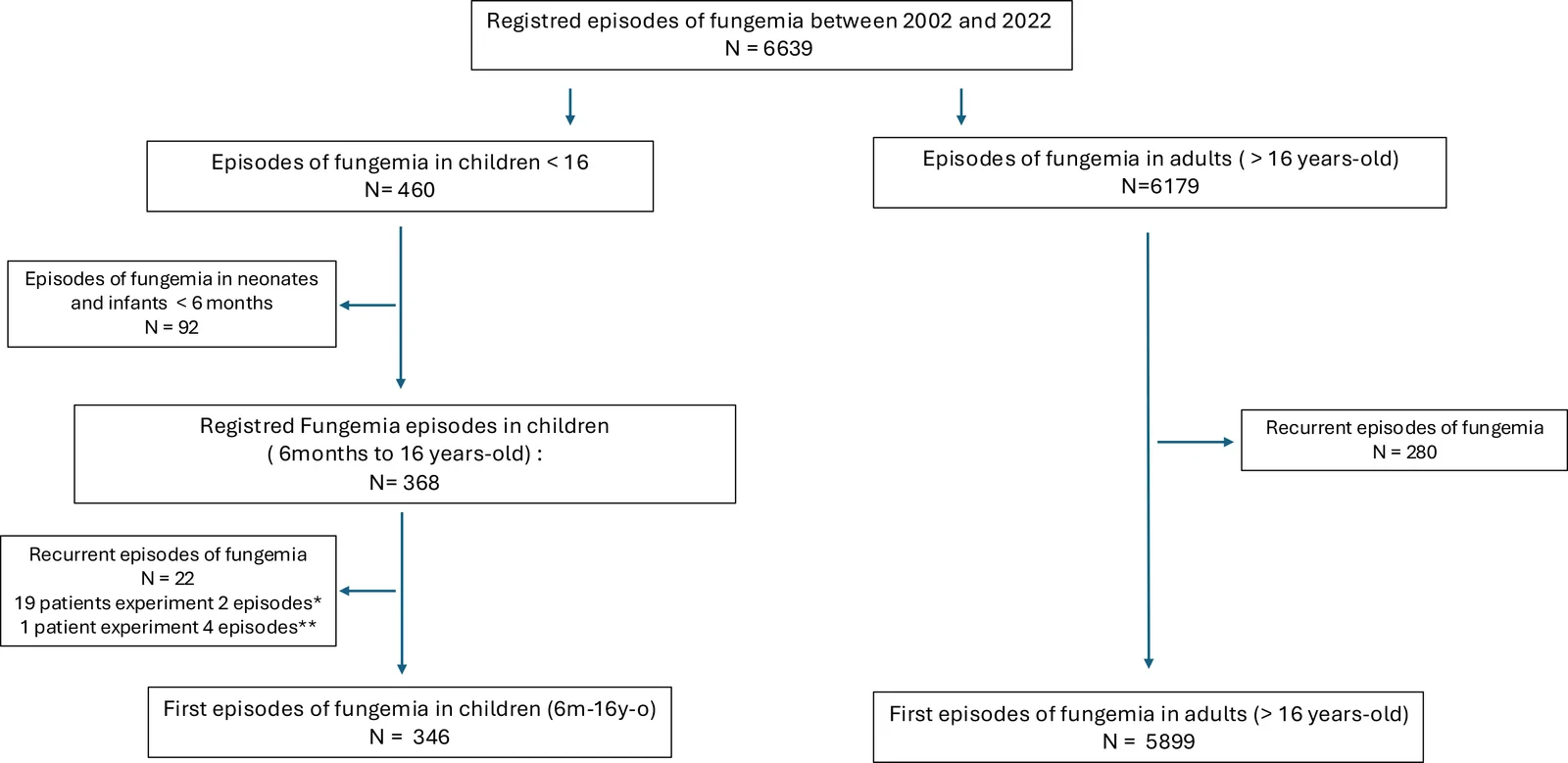
Study profile. *C.*, Candida. ∗1 patient had 2 episodes occurring seven years apart (*C. albicans and C. parapsilosis*); 18 patients had 2 episodes less than six months apart. ∗∗1 patient had 4 episodes within 2 months (*C. lusitaniae*).

This study was based on data from the YEASTS program, a prospective, multicenter, hospital-based surveillance study of patients with bloodstream yeast infections. The program was launched in 2002 in the Paris area and involves 27 university hospitals, including 9 centers providing specialized pediatric care. All patients with at least one blood culture positive for yeast were eligible for inclusion. Among patients with multiple episodes of yeast bloodstream infection recorded in the database, only the first (incident) episode occurring between October 1, 2002, and December 31, 2022, was considered for the present analysis. Episodes occurring in patients aged 6 months through 16 years were included in the paediactric population (children), while episodes concerning patients above 16 years of age were included in the adult population (adults) (see Study profile in Fig. 1).

Clinical and epidemiological data were prospectively collected using a standardized electronic case report form (eCRF) developed on the VOOZANOO™ platform (http://www2.voozanoo.net/) (for more information, see Supplemental Material). To describe the epidemiology of yeast bloodstream infections in children, we analyzed demographic characteristics, underlying risk factors, intensive care unit (ICU) stay at diagnosis, species distribution, antifungal susceptibility patterns, previous antifungal exposure, first-line antifungal therapy, and 30-day all-cause mortality.

### Isolates

All clinical isolates were sent to the National Reference Center for invasive Mycoses and Antifungals (NRCMA) and identified at the species level using process previously described^7^ (for more information, see Supplemental Material). For all isolates, Minimum Inhibitory Concentrations (MICs) for fluconazole, voriconazole, posaconazole and flucytosine were determined, according to the EUCAST broth microdilution standardized method.^19^ Additionally, caspofungin, micafungin and amphotericin B MICs were determined with a modified version of the protocol (briefly, antifungal agents were diluted in AM3 medium instead of RPMI medium, as previously described^8^). MIC50 and MIC90 were respectively calculated for each species as values inhibiting at least 50% and 90% of their isolates.

When available, MIC breakpoints,^20^ ECOFF^21^ or proposal values determined for rare yeasts^22^ were used to determine the percentage of resistant or non-wild-type (NWT) isolates, respectively. These values are summarized in Supplemental Table S1. *P. kudriavzevii* (syn. *C. krusei*) was considered as intrinsically resistant to fluconazole and *T. asahii* was considered as intrinsically resistant to echinocandins. *T. asahii* isolates having MIC > 16 mg/L for fluconazole are considered as NWT.

According to a previous study, caspofungin MIC threshold of 0.25 mg/L was used to distinguish between wild-type and non-wild-type isolates of *C. albicans, C. tropicalis, N. glabratus (syn. C. glabrata), P. kudriavzevii (syn. C. krusei), Candida lusitaniae, Kluyveromyces marxianus (syn. Candida kefyr)* and *Candida dubliniensis*.^8^ For amphotericin B and flucytosine, MIC90 values determined are used as threshold to identify putative NWT isolates.

### Definitions

Considering the high diversity of conditions, and to provide a description relevant for clinical practice and health policy makers, hierarchical ranking based on expert knowledge was used to assign one principal risk factor to each patient. Risk-factor ranking started with hematological diseases associated with hematologic stem cell transplantation (HSCT), hematological malignancies not associated with HSCT (HM), solid tumors, solid organ transplantations (SOT), surgery within 30 days and other diseases (Other). Thus, a case-patient with HM and solid tumor was recorded as HM.

“Previous antifungal treatment” was defined as antifungal treatment at any time during the 30 days preceding the fungemia, whereas “first-line antifungal treatment” was defined as antifungal treatment started at the time of the positive blood culture.

### Reporting

STROBE guidelines were followed.

### Ethics

The research described herein was carried out in compliance with French law and the Declaration of Helsinki (as adopted in 2000), and was approved by the Institut Pasteur institutional review board (IRB #2009-34). Approval of the “Commission Nationale de l’Informatique et des Libertés” was obtained, ensuring that patients’ data were kept anonymous according to French regulations. As the data are collected for surveillance purposes, written consent is not mandatory.

### Statistical analyses

This study being exploratory and aiming at characterizing the underlying epidemiology of pediatric yeast infections, no sample size determination nor power requirement was conducted prior to data analysis. Characteristics of the pediatric population were evaluated using univariable statistical methods. Categorical variables were analyzed using either the Chi-square test or Fisher’s exact test, as appropriate, while continuous variables were assessed using the non-parametric Mann–Whitney U test. Although they provide slightly less statistical power than their parametric counterpart, non-parametric tests were favored to provide a consistent framework for all descriptive analyses, accommodating possibly imbalanced or small subgroups when partitioning the population by *e.g.,* groups of causative strain or primary risk factor. Mixed infections, defined as fungemia episodes involving multiple yeast species simultaneously, were excluded from the analyses describing patient characteristics according to species involved. Associations between clinical outcome (1-month survival) and clinical covariates were examined through logistic regression with complete case analyses. Missing data are reported whenever applicable and no imputation method was used. A single multivariable logistic regression model was constructed, incorporating covariates selected *a priori* based on existing literature or clinical expertise. In regression approaches, mixed-effect models were used to assess the intra-class correlation coefficient whenever clustering by center could be expected. When such component was relevant, a random effect was included in the model to jointly model within-center and between-center variance. Kaplan–Meier survival curves stratified by causative strains were compared using the log-rank test. Finally, comparisons between adult and pediatric populations were performed using both univariable and multivariable approaches, including χ^2^ tests for categorical variables and Wilcoxon rank-sum tests for continuous variables. As a sensitivity analysis, when comparing clinical features across data subset, we ensured significant results did not arise from differential missing data patterns by checking that missing data did not change the outcome when coded as a dedicated category or discarded. In case patients had multiple episodes, only the first one was included in the analysis therefore discarding the few episodes that could have involved competition between strains: as such, no longitudinal approaches were used. All statistical analyses were conducted using the R software (v4.4.2)^23^ as well as packages tableone (v0.13.2), lme4 (v2.0-1), performance (v0.17.0) and survival (v3.8–6).

### Role of the funding source

Institut Pasteur and Santé Publique France did not have any role in study design, collection, analysis, interpretation of the data, writing the report or decision to submit the paper for publication.

## Results

A total of 368 episodes of fungaemia were recorded from 346 children between October 2002 and December 2022. Only the first incident episode from each patient was analyzed thus excluding 22 additional episodes from 20 patients (see Study profile in Fig. 1). Characteristics of the children are summarized in Table 1. The sex ratio (M/F) was 60.1%, and the median age at the time of fungemia was 5.85 ± 4.99 years. Hematological malignancy (HM) was the most common main risk factor for fungemia, affecting 20.5% of children (n = 71), followed by recent surgery (n = 67; 19.4%), solid tumor (n = 45; 13%), HSCT (n = 24; 6.9%) and SOT (n = 12; 3.5%). At fungaemia diagnosis, 34% of children (n = 119) were hospitalized in ICU, and most of them (n = 295, 91.6%) had a central venous catheter. Only 16.5% of children (n = 57) were pre exposed to antifungal, more frequently with echinocandins after 2012 (p = 0.045) (Table 1). Children characteristics varied according to the main risk factor for fungemia and children’s age (Tables 2 and 3); children with HSCT, HM and SOT were older (p < 0.001) and were more likely to have central venous catheters (p < 0.001), whereas children with SOT or surgery were more frequently hospitalized in ICU at diagnosis. Pre-exposition to echinocandins was most frequent in children with SOT (n = 3; 25%), HSCT (n = 5; 20.8%) and HM (n = 10; 14.1%) in comparison to children with other risk factors.

**Table 1:** Characteristics of children with fungemia.

	Total n = 346 (%)	2002–2011 n = 152 (%)	2012–2022 n = 194 (%)	p-value
Sex male/female	208/138 (60.1)	96/56 (63.2)	112/82 (57.7)	0.362
Age (years), mean ± SD	5.85 ± 4.99	6.55 ± 5.18	5.31 ± 4.79	**0.022**
Age group				**0.014**
6 months–2 years	125 (36.1)	42 (27.6)	83 (42.8)	
2–5 years	75 (21.7)	33 (21.7)	42 (21.6)	
5–10 years	53 (15.3)	30 (19.7)	23 (11.9)	
>10 years	93 (26.9)	47 (30.9)	46 (23.7)	
Main risk factor for fungemia				0.959
HSCT	24 (6.9)	10 (6.6)	14 (7.2)	
Hematological malignancy	71 (20.5)	33 (21.7)	38 (19.6)	
Solid tumor	45 (13)	20 (13.2)	25 (12.9)	
Solid organ transplantation	12 (34.7)	4 (2.6)	8 (4.1)	
Surgery within 30 days	67 (19.4)	31 (20.4)	36 (18.6)	
Other	127 (36.7)	54 (35.5)	73 (37.6)	
HIV^a^	4/274 (1.5)	3 (2)	1/122 (0.8)	0.776
ICU hospitalization at diagnosis	119 (34.4)	49 (32.2)	70 (36.1)	0.526
Central venous catheter^b^	295/322 (91.6)	139 (91.4)	156/170 (91.8)	1.000
Previous antifungal treatment				0.458
All	57 (16.5)^e^	22 (14.5)	35 (18)	
Azoles	28 (8.1)	17 (11.2)	16 (8.2)	
Fluconazole	24 (6.9)	12 (7.9)	12 (6.2)	
Itraconazole	1 (0.3)	0 (0.0)	1 (0.5)	
Voriconazole	3 (0.9)	0 (0.0)	3 (1.5)	
Echinocandin	23 (6.6)	5 (3.3)	18 (9.3)	**0.045**
Amphotericin B deoxycholate	10 (2.9)	8 (5.3)	2 (1)	**0.024**
L-Amphotericin B	1 (0.3)	0 (0)	1 (0.5)	1.000
Species				
Single pathogen	336 (97.1)	148 (97.4)	188 (96.9)	0.485
*C. albicans*	140 (41.7)	67 (44.1)	78 (40.2)	0.605
*C. parapsilosis*	79 (23.5)	33 (21.7)	47 (24.2)	0.619
*C. tropicalis*	34 (10.1)	18 (11.8)	17 (8.8)	0.255
*C. lusitaniae*	14 (4.2)	3 (2)	12 (6.2)	0.100
*N. glabratus* (syn. *C. glabrata*)	14 (4.2)	8 (5.3)	7 (3.6)	0.448
*K. marxianus* (syn, *C. kefyr*)	9 (2.7)	4 (2.6)	5 (2.6)	1.000
*P. kudriavzevii* (syn. *C. krusei*)	9 (2.7)	3 (2.0)	7 (3.6)	0.564
*Trichosporon asahii*	7 (2)	4 (2.6)	3 (1.5)	0.744
*C. dubliniensis*	6 (1.8)	1 (0.7)	5 (2.6)	0.346
*Other species*^f^	24 (7.1)	11 (7.2)	13 (6.7)	0.884
Multiple pathogen	10 (2.9)	4 (2.6)	6 (3.1)	1.000
First-line of treatment^c^	332/343 (96.8)	148 (97.4)	184/191 (96.3)	0.589
Echinocandin	151/332 (45.4)	49 (32.2)	102/191 (53.4)	**<0.001**
L-Amphotericin B	48/332 (14.5)	27 (17.8)	21/191 (11)	0.072
Triazoles	131/332 (39.5)	72 (47.4)	59/191 (30.9)	**0.002**
5-FC	17/332 (5.1)	10 (6.6)	7/191 (3.7)	0.217
Overall death at 30 days^d^	43/318 (13.5)	17/146 (11.6)	26/172 (15.1)	0.461

SD, standard deviation; HCST, hematopoietic stem cell transplantation; HIV, human immunodeficiency virus; ICU, Intensive Care Unit; *C.*, Candida; *N.*, Nakaseomyces; *K.*, Kluyveromyces; *P.*, Pichia; 5-FC, flucytosine.

The Results that are shown in bold are statistically significant.

a 72 missing data.

b 24 missing data.

c 3 missing data.

d 28 missing data.

e Including 5 dual therapies (amphotericin B deoxycholate + fluconazole (n = 3); echinocandin + triazole (n = 2)).

f Described in Supplemental Table S2.

**Table 2:** Characteristics of children with fungemia according to the main risk factor.

	HSCT (n = 24)	HM (n = 71)	Solid cancer (n = 45)	SOT (n = 12)	Surgery (n = 67)	Other (n = 127)	p-value
Sex (Male/Female)	13/11 (54.2)	43/28 (60.6)	22/23 (48.9)	7/5 (58.3)	38/29 (56.7)	85/42 (66.9)	0.355
Age, mean ± SD	8.2 (5.1)	7.8 (5.1)	5.7 (4.5)	7.5 (4.6)	5.8 (5.2)	4.3 (4.5)	**<0.001**
Age group							**<0.001**
6 months–2 years	4 (16.7)	13 (18.3)	12 (26.7)	1 (8.3)	28 (41.8)	67 (52.8)	
2–5 years	5 (20.8)	17 (23.9)	16 (35.6)	5 (41.7)	11 (16.4)	21 (16.5)	
5–10 years	6 (25.0)	12 (16.9)	6 (13.3)	1 (8.3)	9 (13.4)	19 (15.0)	
>10 years	9 (37.5)	29 (40.8)	11 (24.4)	5 (41.7)	19 (28.4)	20 (15.7)	
HIV^c^	0/20 (0)	1/65 (1.5)	0/42 (0)	0/9 (0)	0/48 (0)	3/90 (3.3)	0.555
CVC^d^	24 (100)	68/69 (95.6)	43/43 (100)	10/11 (90.9)	54/62 (87.1)	96/113 (85)	**0.002**
ICU	3 (12.5)	9 (12.7)	6 (13.3)	7 (58.3)	36 (53.7)	58 (45.7)	**<0.001**
Previous antifungal treatment^a^	10 (41.7)	17 (23.9)	4 (8.9)	4 (33.3)	7 (10.4)	15 (11.8)	**<0.001**
Azoles	2 (8.3)	4 (5.6)	3 (6.6)	1 (8.3)	6 (9)	12 (9.4)	0.947
Echinocandin	5 (20.8)	10 (14.1)	0 (0.0)	3 (25)	2 (3)	3 (2.4)	**<0.001**
Amphotericin B deoxycholate	3 (12.5)	2 (2.8)	1 (2.2)	0 (0.0)	2 (3)	2 (1.6)	0.177
L-Amphotericin B	0 (0)	1 (1.4)	0 (0)	0 (0)	0 (0)	0 (0)	0.642
Species							**<0.001**
*C. albicans*	8 (33.3)	14 (19.7)	21 (46.7)	6 (50.0)	37 (55.2)	60 (47.2)	**<0.001**
*C. parapsilosis*	2 (8.3)	13 (18.3)	13 (28.9)	4 (33.3)	11 (16.4)	40 (31.5)	**0.039**
*C. tropicalis*	3 (12.5)	14 (19.7)	5 (11.1)	0 (0.0)	6 (9.0)	9 (7.1)	0.090
*C. lusitaniae*	2 (8.3)	4 (5.6)	2 (4.4)	0 (0.0)	4 (6.0)	3 (2.4)	0.641
*N. glabratus* (syn. *C. glabrata*)	4 (16.7)	1 (1.4)	1 (2.2)	2 (16.7)	4 (6.0)	4 (3.1)	**0.010**
*K. marxianus* (syn. *C. kefyr*)	0 (0.0)	9 (12.7)	0 (0.0)	0 (0.0)	0 (0.0)	0 (0.0)	**<0.001**
*P. kudriavzevii* (syn. *C. krusei*)	2 (8.3)	5 (7.0)	1 (2.2)	1 (8.3)	1 (1.5)	0 (0.0)	**0.029**
*C. dubliniensis*	1 (4.2)	0 (0.0)	0 (0.0)	0 (0.0)	2 (3.0)	3 (2.4)	0.549
*T. asahii*	0 (0.0)	3 (4.2)	1 (2.2)	0 (0.0)	1 (1.5)	2 (1.6)	0.744
Other species^b^	2 (8.3)	9 (12.7)	1 (2.2)	0 (0.0)	4 (6.0)	11 (8.7)	0.330
First-line of treatment^e^	23 (95.8)	71 (100)	42/44 (95.4)	12 (100)	67 (100)	117/125 (93.6)	0.061
Echinocandin	19 (79.2)	38 (53.5)	13/44 (29.5)	6 (50.0)	26 (38.8)	49/125 (39.2)	**0.001**
L-Amphotericin B	7 (29.2)	23 (32.4)	2/44 (4.5)	2 (16.7)	5 (7.5)	9/125 (7.2)	**<0.001**
Triazoles	2 (8.3)	10 (14.1)	23/44 (52.3)	4 (33.3)	39 (58.2)	53/125 (42.4)	**<0.001**
5-FC	1 (4.2)	6 (8.5)	2/44 (4.5)	2 (16.7)	3 (4.5)	3/125 (2.4)	0.165
Overall death at 30 days^f^	3/23 (13.0)	8/67 (11.9)	3/44 (6.8)	3/11 (27.3)	9/46 (16.1)	17/117 (14.5)	0.545

SD, standard deviation; HIV, human immunodeficiency virus; CVC; central veinous catheter; ICU, Intensive Care Unit; *C.*, Candida; *N.*, Nakaseomyces; *K.*, Kluyveromyces; *P.*, Pichia; 5-FC, flucytosine.

The Results that are shown in bold are statistically significant.

a Dual therapy in 5 patients.

b Described in Supplemental Table S2.

c 72 missing data.

d 24 missing data.

e 3 missing data.

f 28 missing data.

**Table 3:** Characteristics of children with fungemia according to childrens’ age.

	6 months −2 years (n = 125)	2–5 years (n = 75)	5–10 years (n = 53)	>10 years (n = 93)	p-value
Sex (Male/Female)	80/45 (64)	46/29 (61.3)	33/20 (62.3)	49/44 (52.7)	0.380
Main risk factor for fungemia					**<0.001**
HSCT	4 (3.2)	5 (6.7)	6 (11.3)	9 (9.7)	
Hematological malignancy	13 (10.4)	17 (22.7)	12 (22.6)	29 (31.2)	
Solid cancer	12 (9.6)	16 (21.3)	6 (11.3)	11 (11.8)	
Solid organ transplantation	1 (0.8)	5 (6.7)	1 (1.9)	5 (5.4)	
Surgery within 30 days	28 (22.4)	11 (14.7)	9 (17)	19 (20.4)	
Other	67 (53.6)	21 (28)	19 (35.8)	20 (21.5)	
HIV^c^	1/80 (1.3)	1/64 (1.6)	1/47 (2.1)	1/83 (1.2)	0.999
CVC^d^	105/111 (94.6)	68/72 (90.7)	44/50 (88)	78/89 (87.6)	0.191
ICU	62 (49.6)	16 (21.3)	13 (24.5)	28 (30.1)	**<0.001**
Species					**0.002**
*C. albicans*	53 (42.4)	33 (44)	24 (45.3)	36 (38.7)	0.788
*C. parapsilosis*	40 (32)	18 (24)	10 (18.9)	15 (16.1)	**0.043**
*C. tropicalis*	6 (4.8)	15 (20)	9 (17)	7 (7.5)	**0.002**
*C. lusitaniae*	5 (4)	3 (4)	1 (1.9)	6 (6.5)	0.681
*N. glabratus* (syn. *C. glabrata*)	4 (3.2)	0 (0)	4 (7.5)	8 (8.6)	**0.019**
*K. marxianus* (syn. *C. kefyr*)	1 (0.8)	2 (2.7)	1 (1.9)	5 (5.4)	0.196
*P. kudriavzevii* (syn. *C. krusei*)	1 (0.8)	0 (0)	2 (3.8)	7 (7.5)	**0.008**
*C. dubliniensis*	1 (0.8)	1 (1.3)	1 (1.9)	3 (3.2)	0.562
*T. asahii*	4 (3.2)	1 (1.3)	0 (0)	2 (2.2)	0.735
Other species^a^	13 (10.4)	4 (5.3)	3 (5.7)	7 (7.5)	0.601
Previous antifungal treatment^b^	17 (13.6)	11 (14.7)	11 (20.8)	18 (19.4)	0.533
Azoles	11 (8.8)	7 (9.3)	5 (9.4)	5 (5.4)	0.693
Echinocandin	6 (4.8)	2 (2.7)	5 (9.4)	10 (10.8)	0.122
Amphotericin B deoxycholate	2 (1.6)	3 (4)	2 (3.8)	3 (3.2)	0.702
L-Amphotericin B	0 (0)	0 (0)	0 (0)	1 (1.1)	0.639
First-line of treatment^e^	119/123 (96.7)	73/74 (98.6)	51 (96.2)	89 (95.7)	0.763
Echinocandin	50/123 (40.7)	31/74 (41.9)	27 (50.9)	43 (46.2)	0.593
L-Amphotericin B	15/123 (12.2)	16/74 (21.6)	7 (13.2)	10 (10.8)	0.220
Triazoles	55/123 (44.7)	24/74 (32.4)	14 (26.4)	38 (40.9)	0.304
5-FC	1/123 (0.8)	5/74 (6.7)	5 (9.4)	6 (6.5)	**0.019**
Overall death at 30 days^f^	18/112 (16)	8/72 (11.1)	5/47 (10.6)	12/87 (13.8)	0.765

HCST, hematopoietic stem cell transplantation; HIV, human immunodeficiency virus; CVC, central veinous catheter; ICU, Intensive Care Unit; *C.*, Candida; *N.*, Nakaseomyces; *K.*, Kluyveromyces; *P.*, Pichia; *T.*, Trichosporon; 5-FC, flucytosine.

The Results that are shown in bold are statistically significant.

a Described in Supplemental Table S2.

b Dual therapy in 5 patients.

c 72 missing data.

d 24 missing data.

e 3 missing data.

f 28 missing data.

**Fig. 2:**
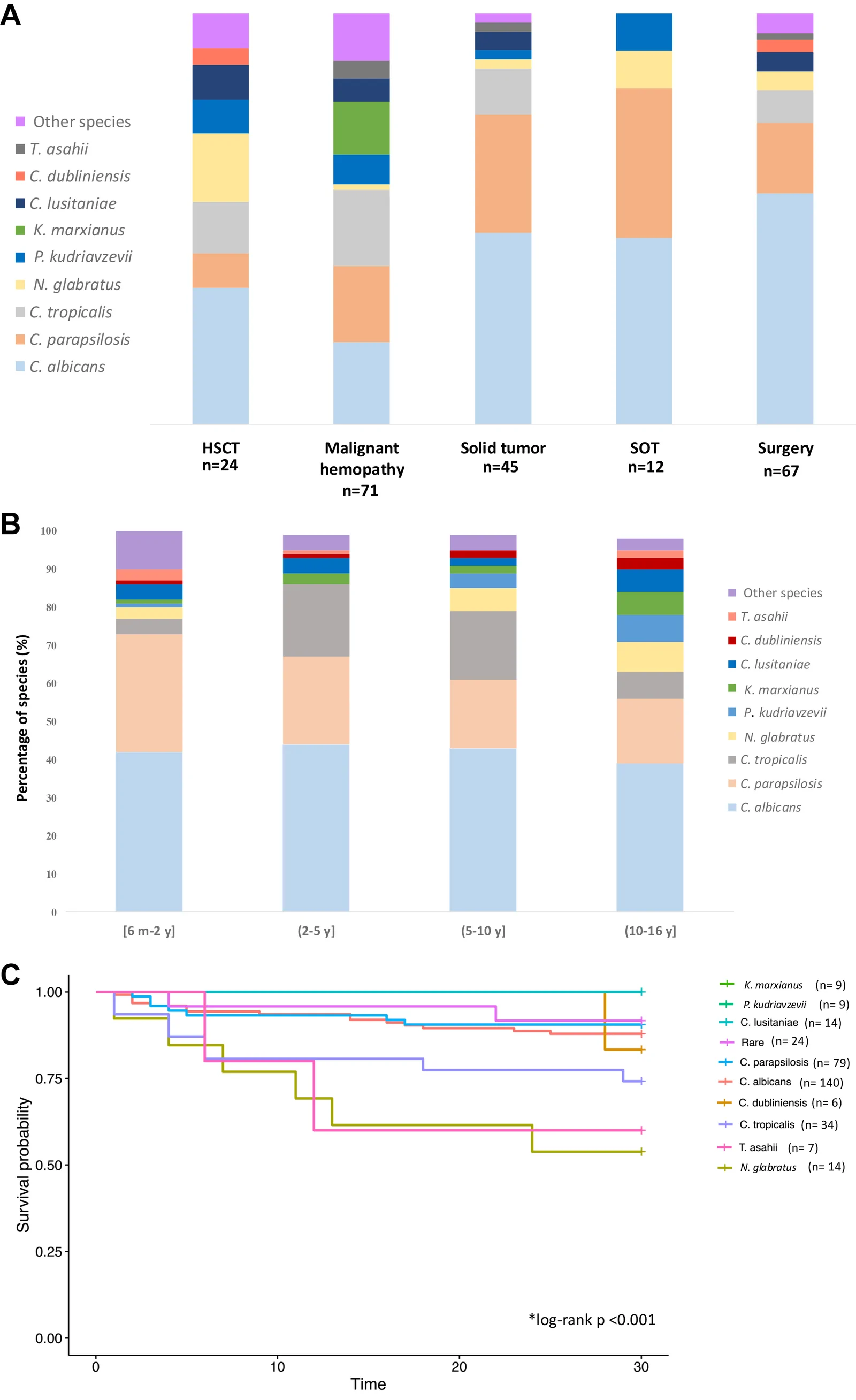
**(A)** Species distribution according to the main risk factors for fungemia. **(B)** Species distribution according to children’s age groups. **(C)** Overall 30-day mortality of fungemia according to the species involved. Differences in 30-day survival were assessed by the log-rank test. ∗*C.*, Candida; *N.*, Nakaseomyces; *K.*, Kluyveromyces; *P.*, Pichia; *T.*, Trichosporon; HCST, hematopoietic stem cell transplantation; SOT, solid organ transplantation; m, months; y, years.

The most frequent species involved in incident fungemia in children are summarized in Table 1 and Figs. 1 and 2. *C. albicans* was the most frequently isolated species (n = 140; 41.7%), followed by *C. parapsilosis stricto sensu* (n = 79; 23.5%) and *C. tropicalis* (n = 34; 10.1%). *N. glabratus (syn. C. glabrata) was* responsible for only 4% of episodes (n = 14) and *T. asahii* accounted for 2% (n = 7) of the causative species. Other rare species are summarized in Supplemental Table S2. Species distributions varied significantly according to the main risk factor for fungemia (p < 0.001). Involved yeast species were more diverse in patients with hematological diseases (HSCT and HM) (Fig. 2A, Table 2), with *C. albicans* being less frequent, especially in patients with HM (n = 14; 19.7%). *N. glabratus (syn. C. glabrata) was* more frequent in children with HSCT (n = 4; 16.7%) than in those without. *C. parapsilosis* was more frequent in children with SOT (n = 4; 33.3%) and solid cancer and was rare in patients with HSCT (n = 2; 8.3%) and HM (n = 13; 18.3%). *C. tropicalis* was more frequent, and *K. marxianus (syn. C. kefyr)* was observed exclusively in patients with HM. Species distributions also differed significantly according to childrens’ age (p = 0.001); children with *C. parapsilosis* fungemia were younger and children with *P. kudriavzevii (syn. C. krusei)* or *N. glabratus (syn. C. glabrata)* fungemia were older (Table 3, Table 4, Table 5 and Fig. 2B). There were no significant changes in the distribution of yeast species causing fungemia according to periods (Table 1).

**Table 4:** Characteristics of children with fungemia (single pathogen) according to species involved.

	*C*. *albicans* (n = 140)	*C. parapsilosis* (n = 79)	*C. tropicalis* (n = 34)	*C. lusitaniae* (n = 14)	*N. glabratus (C*. *glabrata)* (n = 14)	*K. marxianus (C*. *kefyr)* (n = 9)	*P. kudriavzevii (C*. *krusei)* (n = 9)	*C. dubliniensis* (n = 6)	*T. asahii* (n = 7)	Other^e^ (n = 24)	p
Sex (Male/Female)	93/47 (66.4)	46/33 (58.2)	21/13 (61.8)	6/8 (42.9)	8/6 (57.1)	6/3 (66.7)	6/3 (66.7)	3/3 (50)	4/3 (57.1)	9/15 (37.5)	0.351
Age, mean ± SD	5.72 (4.95)	4.54 (4.72)	6.08 (4.11)	6.38 (5.21)	9.02 (5.64)	9 (5.1)	11.1 (4.26)	8.85 (5.76)	4.66 (5.2)	4.99 (5.07)	**0.001**
Age group											**0.009**
6 months–2 years	51 (36.4)	38 (48.1)	5 (14.7)	5 (35.7)	4 (28.6)	1 (11.1)	1 (11.1)	1 (16.7)	4 (57.1)	12 (50)	–
2–5 years	32 (22.9)	17 (21.5)	14 (41.2)	3 (21.4)	0 (0)	2 (22.2)	0 (0)	1 (16.7)	1 (14.3)	3 (12.5)	
5–10 years	22 (15.7)	9 (11.4)	9 (26.5)	1 (7.1)	3 (21.4)	1 (11.1)	2 (22.2)	1 (16.7)	0 (0)	3 (12.5)	
>10 years	35 (25)	15 (19.0)	6 (17.6)	5 (35.7)	7 (50)	5 (55.6)	6 (66.7)	3 (50)	2 (28.6)	6 (25.0)	
Principal risk factor											**<0.001**
HSCT	8 (5.7)	2 (2.5)	3 (8.8)	2 (14.3)	4 (28.6)	0 (0)	2 (22.2)	1 (16.7)	0 (0)	2 (8.3)	
HM	14 (10)	13 (16.5)	13 (38.2)	4 (28.6)	1 (7.1)	9 (100)	5 (55.6)	0 (0)	3 (42.9)	8 (33.3)	
Solid tumor	21 (15)	13 (16.5)	5 (14.7)	2 (14.3)	1 (7.1)	0 (0)	1 (11.1)	0 (0)	1 (14.3)	1 (4.2)	
SOT	5 (3.6)	4 (5.1)	0 (0)	0 (0)	1 (7.1)	0 (0)	1 (11.1)	0 (0)	0 (0)	0 (0)	
Surgery	36 (25.7)	11 (13.9)	5 (14.7)	3 (21.4)	3 (21.4)	0 (0)	0 (0)	2 (33.3)	1 (14.3)	3 (12.5)	
HIV^a^	3/109 (2.8)	1/58 (1.7)	0/30 (0)	0/11 (0)	0/11 (0)	0/9 (0)	0/8 (0)	0/5 (0)	0/7 (0)	0/19 (0)	0.975
CVC^b^	112/126 (88.8)	68/76 (89.5)	32/32 (100)	14/14 (100)	12/12 (100)	8/8 (100)	8/8 (100)	5/6 (83.3)	6/7 (85.7)	21/23 (91.3)	0.405
ICU	55 (39.3)	23 (29.1)	11 (32.4)	4 (28.6)	4 (28.6)	2 (22.2)	2 (22.2)	4 (66.7)	0 (0.0)	10 (41.7)	0.266
Previous antifungal treatment	20 (14.3)	14 (17.7)	2 (5.9)	0 (0)	6 (42.9)	2 (22.2)	4 (44.4)	1(16.7)	3 (42.9)	4 (16.7)	**0.009**
Azoles	13 (9.3)	6 (7.6)	1 (2.9)	0 (0)	2 (14.2)	0 (0)	2 (22.2)	0 (0)	1 (14.3)	2 (8.3)	0.550
Echinocandin	4 (2.9)	7 (8.9)	1 (2.9)	0 (0)	3 (21.4)	1 (11.1)	2 (22.2)	1 (16.7)	2 (28.6)	2 (8.3)	**0.016**
Amphotericin B	5 (3.6)	1 (1.3)	0 (0)	0 (0)	2 (14.2)	1 (11.1)	0(0)	0 (0.0)	1 (14.3)	1 (4.2)	0.218
First-line of treatment^c^	131/137 (95.6)	76 (96.2)	34 (100)	14 (100)	13 (92.9)	9 (100)	9 (100)	6 (100)	7 (100)	23 (95.8)	0.914
Echinocandin	57/137 (41.6)	28 (35.4)	16 (47.1)	9 (64.3)	8 (57.1)	6 (66.7)	8 (88.9)	4 (66.7)	1 (4.3)	8 (33.3)	**0.015**
Amphotericin B	13/137 (9.5)	11 (13.9)	4 (11.8)	2 (14.3)	4 (28.6)	4 (44.4)	0 (0)	0 (0)	2 (28.6)	6 (25.0)	**0.029**
Triazoles	60/137 (43.8)	31 (39.2)	13 (38.2)	4 (28.6)	2 (14.3)	0 (0)	1 (11.1)	2 (33.3)	5 (71.4)	10 (41.7)	**0.047**
5-FC	4/137 (2.9)	5 (6.3)	1 (2.9)	0 (0)	1 (7.1)	2 (22.2)	0 (0)	1 (16.7)	0 (0)	1 (4.2)	0.209
Overall death at 30 days^d^	15/124 (12.1)	8/75 (10.7)	8/31 (25.8)	0/13 (0)	6/13 (46.2)	0/8 (0)	0/9 (0)	1/6 (16.7)	2/5 (40.0)	2/24 (8.3)	**0.003**

SD, standard deviation; HCST, hematopoietic stem cell transplantation; HM; hematological malignancy; SOT, solid organ transplantation; HIV, human immunodeficiency virus; CVC, central veinous catheter; ICU, Intensive Care Unit; C., Candida; N., Nakaseomyces; K., Kluyveromyces; P., Pichia; T., Trichosporon; 5-FC, flucytosine. The Results that are shown in bold are statistically significant.

a 72 missing data.

b 24 missing data.

c 3 missing data.

d 28 missing data.

e Described in Supplemental Table S2.

**Table 5:** Antifungal susceptibility profile of fungaemia-causing yeasts (single pathogen episodes) and percentage of acquired or intrinsic resistant strains.

	*C. albicans* (n = 140)	*C. parapsilosis* (n = 79)	*C. tropicalis* (n = 34)	*C. lusitaniae* (n = 14)	*N. glabratus (C. glabrata)* (n = 14)	*K. marxianus (C. kefyr)* (n = 9)	*P. kudriavzevii (C. krusei)* (n = 9)	*C. dubliniensis* (n = 6)	*T. asahii* (n = 7)	Total
Caspofungin										
Nbr of strains tested	123/140	72/79	28/34	13/14	10/14	8/9	9/9	6/6	7/7	276
MIC50/MIC90	0.03/0.06	0.25/1	0.03/0.06	0.06/0.06	0.03/0.06	0.03/-	0.125/-	0.015/-	>4/-	–
Range of MIC	<0.007–4	0.06–1	<0.007–2	0.015–0.06	0.03–0.125	<0.007–0.25	0.06–0.125	<0.007–0.03	1 to >4	–
Nbr NWT (%)^a^	2/123 (1.6)	0/72	1/28 (3.5)	0/13 (0)	0/10 (0)	0/8 (0)	0/9 (0)	1/6 (16.7)	7/7 (100)^b^	11/276 (4.0)
Amphotericin B										
Nbr of strains tested	134/140	72/79	33/34	14/14	13/14	8/9	9/9	6/6	7/7	296
MIC50/MIC90	0.06/0.125	0.03/0.125	0.06/0.125	0.125/0.25	<0.125/0.25	0.125/-	0.125/-	<0.015/-	2/-	–
Range of MICs	0.03–0.5	<0.015–0.5	<0.015–0.125	0.03–0.25	0.0.06–0.5	0.06–0.25	0.125–0.25	<0.015–0.125	0.5/>4	–
Nbr NWT (%)^a^	0/134	0/72	0/33	0/14	0/13	0/8	0/9	0/6	0/7	0/296 (0.0)
Fluconazole										
Nbr of strains tested	134/140	72/79	33/34	14/14	13/14	8/9	9/9	6/6	7/7	296
MIC50/MIC90	<0.125/0.25	0.5/1	0.5/1	0.25/1	16/64	0.5/-	32/-	<0.125/-	4/-	–
Ranges of MICs	<0.125–32	0.25–64	<0.125 to >64	<0.125–2	4–64	<0.125–4	32–64	<0.125–0.25	1–32	–
Nbr Resistant or NWT (%)^a^	1/134 (0.7)	2/72 (2.8)	2/33 (6)	0/14 (0)	6/13 (46.2)	2/8 (25)	9/9 (100)^b^	0/6 (0)	2/7 (28.5)	24/296 (8.1)
Voriconazole										
Nbr of strains tested	134/140	72/79	33/34	14/14	13/14	8/9	9/9	6/6	7/7	296
MIC50/MIC90	<0.015/<0.015	<0.015/0.03	0.03/0.125	<0.015/<0.015	0.25/4	<0.015/-	0.5/-	<0.015/-	0.125/-	–
Ranges of MICs	<0.015 to >4	<0.015–1	<0.015 to >4	<0.015–0.06	0.06 to >4	<0.015–0.03	0.25–1	<0.015	0.03–0.5	–
Nbr resistant or NWT (%)^a^	1/134 (0.7)	1/72 (1.4)	2/33 (6)	1/14 (7)	3/13 (23)	0/8 (0)	0/9 (0)	0/6 (0)	NA	8/289 (2.8)
5-FC										
Nbr of strains tested	134/140	72/79	33/34	14/14	13/14	8/9	9/9	6/6	7/7	296
MIC50/MIC90	<0.125/0.5	<0.125/0.25	<0.125/32	<0.125/1	<0.125/<0.125	0.25/-	2/-	<0.125/-	32/-	–
Ranges of MICs	<0.125 to >64	<0.125–0.25	<0.125 to >64	<0.125–32	<0.125	<0.125–8	1–4	<0.125	16 to >64	–
Nbr NWT (%)^a^	12/134 (8.9)	0/72 (0)	3/33 (9.1)	1/14 (7.1)	0/13 (0)	NA	NA	NA	NA	16/266 (6)

*C.*, Candida; *N.*, Nakaseomyces; *K.*, Kluyveromyces; *P.*, Pichia; *T.*, Trichosporon; NWT, non-wild-type; MIC, Minimum Inhibitory Concentrations; 5-FC, flucytosine.

a Defined in the “Material and methods” section.

b Considered as intrinsically resistant.

The proportion of children with prior exposure to echinocandins differed significantly according to the species responsible for fungemia. It was higher in cases of fungemia due to *N. glabratus (syn. C. glabrata)*, *K. marxianus (syn. C. kefyr)*, or *T. asahii* (21.4% (n = 3), 22.2% (n = 2) and 28.6% (n = 2) respectively) compared to fungemia caused by other yeast species (<20%) (p = 0.016) (Table 4).

Resistance profiles are summarized in Table 5. Eleven isolates (4%) were classified as caspofungin-resistant, including four with acquired resistance—2/123 *C. albicans* (1.6%), 1/28 *C. tropicalis* (3.5%), and 1/6 *C. dubliniensis* (16.7%)—and seven with intrinsic resistance, all *T. asahii* (7/7, 100%). Among the four isolates with acquired echinocandin resistance, the S645P amino acid substitution in the HS1 region of the *FKS* gene was identified by Sanger sequencing, as previously described.^8^ Twenty-four isolates (8.1%) were classified as fluconazole-resistant, including 11 with acquired resistance—1/134 *C. albicans* (0.7%), 2/72 *C. parapsilosis* (2.8%), 2/33 *C. tropicalis* (6%), 6/13 *N. glabratus (syn. C. glabrata)* (46.2%), and 2/8 *K. marxianus (syn, C. kefyr)* (25%)—and nine with intrinsic resistance, all *P. kudriavzevii (syn. C. krusei)* (9/9, 100%). For voriconazole and flucytosine, 2.8% (8/289) and 6% (16/266) of isolates, respectively, were classified as non-wild-type (NWT), defined by MIC values above the species-specific clinical breakpoints (BP), ECOFFs, or MIC90. Overall, no isolates were resistant to amphotericin B. We observed that 45.5% (5/11) of patients with caspofungin-resistant yeast fungemia had received an echinocandin within the 30 days preceding fungemia, whereas 12.5% (3/24) of patients with fluconazole-resistant yeast fungemia had received a triazole during the same period (Supplemental Table S3).

At the time of positive blood culture, antifungal treatment was prescribed in 332 episodes of fungemia (96.8%). First-line antifungal treatment included an echinocandin in 45.4% of cases (n = 151), an azole in 39.5% of cases (n = 131), L-Amphotericin B in 14.5% of cases (n = 48) and flucytosine in 5.1% of cases (n = 17) (Table 1). Dual therapy was prescribed in 19 episodes (5.7%). We observed a significant increase in the rate of echinocandin prescriptions as first-line therapy after 2012 (53.4% *vs.* 32.2%, p < 0.001), which was correlated with a significant decrease in the rates of L-Amphotericin B and triazole prescriptions (11% *vs.* 17.8%, p = 0.09 and 30.9% *vs.* 47.4%, p = 0.008, respectively) (Table 1).

First-line antifungal treatment varied according to the principal risk factor for fungemia (Table 2). An echinocandin or amphotericin B was more often prescribed as first-line therapy in children with HSCT, HM or SOT, whereas an azole was preferred as first-line therapy in patients with solid tumors or who had undergone recent surgery (p < 0.001). Flucytosin was almost never prescribed in children under 2 years of age (Table 3).

In seven patients (2%), first-line treatment was inadequate, including two cases of caspofungin-resistant yeast fungemia and five cases of fluconazole-resistant yeast fungemia (Supplemental Table S3).

Overall death at 30 days was 13.5% in children with fungemia (Table 1). It was not different before and after 2012 (Table 1). Female sex, ICU status, *C. tropicalis*, *N. glabratus (syn. C. glabrata)* and *T. asahii* were found to be independently associated with mortality in children with fungaemia (Table 6 and Fig. 2C). Mortality was not associated with age group, antifungal treatment nor the principal risk factor but we observed that the mortality rate among patients with yeast fungemia resistant to caspofungin, fluconazole or voriconazole (50%, 20.8%, 50%) was higher than that observed in the overall population (13.5%).

**Table 6:** Risk factors for death in pediatric patients with incident fungemia due to a single isolate (logistic regression), YEASTS program, Paris area, October 2002 to December 2022.

	Death before day 30 Adj. OR	95% IC	p
Sex male	0.31	0.14–0.67	0.003032^b^
Age categories			0.458140
6 months–2 years	1		
2–5 years	0.43	0.12–1.37	
5–10 years	0.49	0.13–1.63	
>10 years	0.52	0.17–1.62	
Principal risk factor			0.649807
HSCT	0.73	0.09–4.47	
Malignant hemopathy	1.26	0.34–4.48	
Solid tumor	0.56	0.11–2.26	
SOT	3.11	0.46–18.7	
Surgery	0.77	0.25–2.20	
Other	1		
ICU	**3.93**	**1.66–9.83**	**0.001593**^b^
CVC	1.19	0.25–9.66	0.829610
Species			
*C. albicans*	3.32	0.72–24.44	0.130897
*C. parapsilosis*	2.29	0.45–17.60	0.331828
*C. tropicalis*	**13.01**	**2.19–115.72**	**0.003818**^b^
*C. lusitaniae*	NE^a^	NA	0.312976
*N. glabratus* (syn. *C. glabrata*)	**22.81**	**3.23–230.44**	**0.001342**^b^
*K. marxianus* (syn. *C. kefyr*)	NE^a^	NA	0.262531
*P. kudriavzevii* (syn. *C. krusei*)	NE^a^	NA	0.486151
*C. dubliniensis*	2.82	0.09–50.25	0.503704
*Trichosporon asahii*	**31.67**	**2.18–520.91**	**0.012897**^c^
Previous antifungal treatment	1.41	0.47–3.91	0.521803
First-line of treatment			
Echinocandin	0.59	0.21–1.63	0.307494
Amphotericin B	1.19	0.33–1.63	0.788332
Triazoles	0.67	0.23–1.98	0.464205
5-FC	2.36	0.42–10.42	0.301372

HCST, hematopoietic stem cell transplantation; SOT, solid organ transplantation; HIV, human immunodeficiency virus; CVC, central veinous catheter; ICU, Intensive Care Unit; *C.*, Candida; *N.*, Nakaseomyces; *K.*, Kluyveromyces; *P.*, Pichia; 5-FC, flucytosine.

The Results that are shown in bold are statistically significant.

a OR non-estimated due to complete absence of events.

b 0.01.

c 0.05.

A total of 6179 episodes of fungaemia were recorded from 5889 adults during the study period. Principal risk factors for fungemia were significantly different between paediactric and adult population (p < 0.001). Patients with HSCT and HM were more represented in the paediactric population whereas patients with solid tumors and solid organ transplantation were less represented (Table 7). Regarding incident episodes of fungemia, multivariable analysis showed that species distribution, prior antifungal exposure, first-line antifungal therapy, and 30-day mortality differed significantly between pediatric and adult populations, independently of the main risk factors for fungemia. *N. glabratus* (*syn. C. glabrata*) was less frequently observed in children, whereas *C. parapsilosis* and *T. asahii* were more common. Children also had a higher rate of prior antifungal exposure, and amphotericin B was prescribed significantly more often than in adults. Overall 30-day mortality was significantly lower in children, approximately 3.7-fold lower than in adults (13.5% *vs.* 40.9%, p < 0.001) (Table 7).

**Table 7:** Comparison of children and adult’s characteristics.

	Children n = 346 (%)	Adults n = 5889 (%)	p	OR	95% IC	p
Sex (Male/Female)	208/138 (60.1)	3616/2273 (61.4)	0.674	1.19	0.84–1.69	0.3372
Age, mean ± SD	5.85 ± 4.99	60.53 ± 16.76	**< 0.001**			
Principal risk factor for fungemia			**<0.001**			
HSCT	24 (6.9)	125 (2.1)		**0.36**	**0.15–0.88**	**0.0247**
Hematological malignancy	71 (20.5)	714 (12.1)		**0.48**	**0.27–0.83**	**0.0093**
Solid tumor	45 (13)	1880 (31.9)		**0.35**	**0.2–0.62**	**0.0003**
Solid organ transplantation	12 (3.5)	350 (5.9)		**0.4**	**0.18–0.9**	**0.0277**
Surgery within 30 days	67 (19.4)	1052 (17.9)		1.29	0.76–2.17	0.3466
Other	127 (36.7)	1768 (30)		1		
ICU	119 (34.4)	2714 (46.1)	**<0.001**	0.81	0.55–1.2	0.3016
Central venous catheter	295/322 (91.6)^a^	4264/5512 (77.4)^b^	**<0.001**	**4.3**	**2.14–8.63**	**<0.001**
Previous antifungal treatment	57 (16.5)	652 (11.1)	**<0.001**	1.28	0.78–2.13	0.3303
Pathogen			**<0.001**			
*C. albicans*	146 (42.2)	2898 (49.3)	**0.012**	0.88	0.49–1.55	0.6503
*C. parapsilosis*	83 (24)	651 (11.1)	**<0.001**	**2.27**	1.24–4.18	**0.0082**
*C. tropicalis*	37 (10.7)	498 (8.5)	0.181	1.07	0.52–2.22	0.8509
*C. lusitaniae*	15 (4.3)	111 (1.9)	**0.003**	1.35	0.47–3.91	0.5776
*N. glabratus* (syn. *C. glabrata*)	16 (4.6)	1094 (18.6)	**<0.001**	**0.28**	**0.12–0.65**	**0.0029**
*K. marxianus* (syn. *C. kefyr*)	9 (2.6)	104 (1.8)	0.357	1.62	0.48–5.39	0.4343
*P. kudriavzevii* (syn. *C. krusei*)	10 (2.9)	184 (3.1)	0.930	1.37	0.49–3.85	0.5452
*Trichosporon asahii*	7 (2)	17 (0.2)	**<0.001**	2.32	0.41–13.16	0.3433
*C. dubliniensis*	6 (1.7)	85 (1.4)	0.837	1.47	0.43–5.01	0.5379
First-line of treatment	332/343 (96.8)^c^	4989/5864 (85.1)^d^	**<0.001**			
Echinocandin	151/343 (44)	2275/5864 (38.8)	0.054	1.17	0.68–2.02	0.5669
Amphotericin B	48/343 (14)	294/5864 (5)	**<0.001**	**2.08**	**1.04–4.14**	**0.0376**
Triazoles	131/343 (38.2)	2241/5864 (38.2)	0.993	1.36	0.79–2.32	0.2628
5-FC	17/343 (5)	197/5864 (3.4)	0.115	2.37	0.96–5.87	0.0609
Overall death at 30 days	43/318 (13.5)^e^	2194/5358 (40.9)^f^	**<0.001**	**0.27**	**0.17–0.43**	**<0.001**

SD, standard deviation; HCST, hematopoietic stem cell transplantation; ICU, Intensive Care Unit; CVC, central veinous catheter; *C.*, Candida; *N.*, Nakaseomyces; *K.*, Kluyveromyces; *P.*, Pichia; 5-FC, flucytosine.

The Results that are shown in bold are statistically significant.

a 24 missing data.

b 377 missing data.

c 3 missing data.

d 25 missing data.

e 28 missing data.

f 531 missing data.

## Discussion

Using data from the YEASTS surveillance program, we report here the epidemiology of yeast bloodstream infections in children and adolescents, excluding neonates, in the Paris area. This unique database has enabled the centralization of clinical and microbiological data that has been reliably monitored and analyzed over a 20-year period during which therapeutic and diagnostic practices have remained broadly similar in France. In our study, malignant hemopathy (non-HSCT) was the main risk factor for fungemia, affecting 20.5% of children, followed by solid tumor (13%), recent surgery (19.4%), HSCT (6.9%) and SOT (3.5%), while only 1.5% of children were HIV-infected. Thirty-four percent of patients were hospitalized in ICU at the onset of fungemia. These results are quite different to the findings of a retrospective multicenter study conducted in 10 European countries that reported 1395 episodes of candidemia, in which 36.4% occurring in neonates. Indeed, in this study, children with surgery and children with a malignancy were less represented (representing 11.9% and 26.3% of the non-neonatal population respectively). However, the proportion of patients in the ICU at the time of fungemia was similar (representing 33% of the non-neonatal population).^10^ Notably, the vast majority of patients (91.6%) had a central venous catheter in place, a finding consistent with previously published data of fungemia in non-neonatal pediatric populations.^12^^,^^16^

In our paediactric population, *C. albicans* was the most frequent species (42%), however more than 50% of fungemia were caused by non-albicans *Candida* (NAC) or other yeast species with *C parapsilosis* (23.5%) and *C. tropicalis* (10%) as the most frequent NAC. These results are in line with recent studies conducted in non-neonatal paediactric populations, reporting NAC accounted for 48, 55, 61 or 65% of species involved in Chile, Canada, US or Thailand, respectively.^11^, ^12^, ^13^^,^^16^ Distribution of NAC also varied depending on the country, with *C. parapsilosis* and *C. tropicalis* most frequent in US, Thailand and Chile,^11^^,^^12^ whereas *N. glabratus (syn. C. glabrata)* was more prevalent than *C. tropicalis* in the Canadian study.^13^ In Europe, the retrospective multicenter study conducted in 10 European countries found that *C. albicans* accounted for 48% of isolated species, followed by *C. parapsilosis* and *N. glabratus (syn. C. glabrata)*. As in our study, children admitted to hematology-oncology wards presented the highest rates of NAC species.^10^

We found that *T. asahii* accounted for 2% of causative species of fungemia in paediactric population, mainly children with cancer, *i.e.,* 10 times more than in the adult population. This finding could maybe due to colonization frequency of *T. asahii* which apparently increased with age up to 13–15 years in male and 30–39 years in female patients, subsequently decreasing gradually in both sexes until senescence.^24^ Moreover, it was found to be independently associated with mortality in pediatric population with a mortality rate of 40%. Majority of reported pediatric cases involves neonates or children having hematological malignancies with mortality rates range from 12.5%–58%, this rate varies according to the antifungal agent used as first-line treatment.^25^, ^26^, ^27^ At the time of the fungemia, 28.6% of patients had a history of echinocandin exposure in the 30 days prior to diagnosis. This percentage was significantly higher than in other types of fungemia. Given the intrinsic resistance of all *Trichosporon* species to echinocandins, this raises concerns regarding the selection pressure exerted by echinocandins when used as a preventive strategy in these patients as shown by Lortholary et al.^28^ However, despite the increased use of echinocandins after 2012, the rate of *T. asahii* fungemia was stable during all the time of the study.

In our paediactric cohort, 4% of isolates were resistant to caspofungin (intrinsic or acquired resistance) and 8.1% were resistant to fluconazole. Prior exposure to echinocandins is a recognized risk factor for the development of bloodstream infections caused by yeast species that are intrinsically resistant to this antifungal class.^29^ Furthermore, our data show that 3/4 cases involving isolates with acquired echinocandin resistance (related to FKS mutation) occurred in patients who had previously been preexposed to these agents. Consequently, a known history of echinocandin exposure should prompt rapid species identification and antifungal susceptibility testing to verify that echinocandins remain an appropriate therapeutic option. Interestingly, we did not observe any *N. glabratus (syn. C. glabrata)* isolate resistant to echinocandins, whereas increasing resistance appears to be emerging in the adult population.^8^ Thirteen isolates had an acquired resistance to fluconazole (0.7% of *C. albicans*, 2.8% of *C. parapsilosis*, 6% of *C. tropicalis*, 46.2% of *N. glabratus (syn. C. glabrata)* and 25% of *K. marxianus (syn. C. kefyr)*). Prior azoles exposure in the 30 days preceding fungemia was only observed in 12.5% of cases suggesting that the proportion of resistant strains is rising in environmental settings. This phenomenon is also reported in other studies.^28^

Interestingly, the overall 30-day mortality rate was 13.5% in our paediactric population. This rate is consistent with those reported in recent non-neonatal paediactric candidemia studies from different geographical settings, including cohorts from Chile, Thailand and other countries, in which mortality ranged from 8% to 28.5%.^11^, ^12^, ^13^^,^^16^ These findings suggest that mortality associated with paediactric candidemia remains substantial across diverse healthcare settings despite geographical differences. In our multivariable analysis, female sex, ICU stay, and infection due to *C. tropicalis*, *N. glabratus* (syn. *C. glabrata*), or *T. asahii* were independently associated with 30-day mortality. ICU admission was similarly associated with mortality in both the Chilean and Thai paediactric cohorts.^11^^,^^12^ In contrast, the associations observed with female sex and specific fungal species have not been consistently reported in previous paediactric studies. To our knowledge, data regarding the impact of *Candida* species on mortality in paediactric candidemia remain limited, and results across studies are inconsistent. In the YEASTS program French adult cohort which reported 2507 cases of candidemia published in 2014, authors reported higher mortality rates in cases of *P. kudriavzevii (syn. C. krusei)* and *C. tropicalis* compared with *C. albicans* candidemia in non-ICU population.^18^ However, in a European cohort study of 170 adult patients published in 2024, authors reported higher mortality rates in cases of *C. tropicalis* or *N. glabratus (syn. C. glabrata)* candidemia compared with *C. albicans* or *C. parapsilosis* candidemia (63.6% and 23.7% *vs.* 7.7% and 7.7% respectively), suggesting that these candidemia can be more severe.^30^

When we compared the two periods of our study (before and after 2012), we observed a shift in the age distribution of patients, with a higher proportion of young children in the more recent period. However, we cannot draw definitive conclusions about the cause of this shift, as our study protocol does not allow for the estimation of population-level incidence. Furthermore, consistent with the known influence of age on disease presentation, we observed age-related differences in both risk factor profiles and species distribution. However, despite these temporal changes in patient characteristics, the overall distribution of fungal species and the 30-day mortality rate remained stable between the two periods, suggesting that there has been no major change in the epidemiology or outcomes of pediatric fungemia over time.

Using multivariable analysis, we compared clinical characteristic of the yeast fungemia among adult and pediatric population recovered from the same surveillance program, which has rarely been done. Independently of underlying conditions, we demonstrated that species distribution and mortality differed significantly between the adult and pediatric populations. As in the Chilean study which has also compared data from 384 episodes of candidemia among neonates (n = 25), infants (n = 45), children (n = 64), adults (n = 102), and elderly individuals (n = 148), risk factors, species involved, first-line of treatment and outcomes differed significantly between children and adults. In this study, *N. glabratus (syn. C. glabrata)* was highly prevalent in the adult population compared with the pediatric population, while the opposite was observed for *C. tropicalis* and for *C. lusitaniae*. The overall 30-day survival was 74.2%, significantly higher in infants (82%) and children (86%) compared to neonates (72%), adults (71%) and the elderly (70%).^12^ Beyond the species involved, differences in the severity of underlying diseases and the number of associated comorbidities may partly explain the observed difference in mortality between children and adults. The variation in species distribution responsible for fungemia could be attributed to differences in antifungal use between children and adults, particularly regarding prophylaxis, and possibly to differences in immune system maturity.

This study has several limitations. Indeed, its restriction to the French context limits generalization to other settings and the extended inclusion period (2002–2022) introduces temporal bias, as diagnostic and therapeutic practices evolved considerably over two decades. Furthermore, the participating centers were only found in the Paris area, a very specific metropolitan setting: epidemiology in suburban or remote areas may differ. Finally, these results should be interpreted with great care as they pertain to rare species or resistant isolates which, by nature, are difficult to observe: the sample size is low and therefore statistical power may not be optimal for more complex or subgroups analyses.

While pediatric candidemia has been sporadically documented in multicenter snapshots, the sustained, population-based surveillance presented here fills a critical evidence gap, offering the first reliable incidence trends and risk stratifications for non-neonatal pediatric patients in Europe. Our findings reveal a distinct epidemiological signature: malignant hemopathy emerged as the predominant risk factor (23.6%), a profile that diverges sharply from the surgical predominance observed in adult cohorts and the neonatal intensive care focus of previous pediatric studies. This unique dataset also enabled, within a unified surveillance framework, a direct comparative analysis between pediatric and adult populations -an approach rarely attempted due to the paucity of parallel cohorts. This comparison exposes fundamental age-specific differences in species distribution and outcomes, with a lower mortality rate *i.e.,* almost 4 times lower than in adults, challenging the extrapolation of adult data to pediatric therapeutic strategies.

Moreover, this study establishes pediatric-specific benchmarks for antifungal resistance in this region, with fluconazole resistance at 8.1% and caspofungin resistance at 4%, which is reassuring regarding the efficacy of recommended empirical treatments. However, paediatricians, particularly in oncology, should remain vigilant and consider the possibility of *T. asahii* fungemia in cases of first-line echinocandin treatment failure, as the proportion of *T. asahii* was ten times higher in children than in adults, especially when the child was recently pre-exposed to echinocandins. This study highlights the importance of having pediatric-specific data to better identify at-risk patients and improve their management.

## Contributors

FAL, MDO and FL accessed and verified the underlying data. All authors had final responsibility for the decision to submit for publication. OL contributed to the design and development of the database. FAL, OL, MDO and FL conceptualized the study and FL supervised the study. FAL and TO developed and carried out the statistical analysis plan. MGM, MEB, PM, JG, KBS, FB, EC and The French Mycoses study group participated in recruitment of patients and actively participated in the data collection. KSB coordinated the data collection. FAL, MDO and FL interpreted the data. FAL, MDO and FL developed the first draft of the manuscript. All authors contributed to the review and amendments of the manuscript for important intellectual content and approved this final version for submission.

## Data sharing statement

We support data sharing of the deidentified participant data in accordance a with FAIR principles. Request for access should be directed beginning 3 months and ending 1 year after publication to Fanny Lanternier (fanny.lanternier@pasteur.fr). These proposals will be reviewed and approved on the basis of scientific merit and in accordance with the principles of Institut Pasteur Ethics Charter (https://www.pasteur.fr/sites/default/files/rubrique_linstitut_pasteur/nos_engagements/ethique/ethics_charter_pasteur-2022.pdf). The request concerns the deidentified participant data that underlie the results reported in this article, after de-identification (text, tables, figures, and appendix), and software code. Data will be available at the CNRMA in Institut Pasteur, Paris, France. To gain access, data requesters will need to sign a data access agreement and use the secure data sharing tools provide by Institut Pasteur.

## Declaration of interests

All other authors declare no competing interests.

## Appendix A Supplementary data

Supplementary material and Tables S1–S3

The French Mycoses Group_supp file
