# Supplementary material for: Pediatric yeast fungaemia species patterns, resistance profiles, and outcomes: a 20-year surveillance study in Paris, France
Source: eClinicalMedicine. 2026 Sep 1;99:104177. doi: 10.1016/j.eclinm.2026.104177 (PMC13571122; doi:10.1016/j.eclinm.2026.104177)
Supplement: Supplementary file 1 [file mmc1.pdf]

Pediatric yeast fungaemia species patterns, resistance profiles, and outcomes: a 20-year surveillance study in Paris, France

Supplementary data

Contents list:

- **Supplementary material**
    - Case Report Form (CRF) used in the YEASTS surveillance programme
    - Methods for species identification, antifungal susceptibility, FKS sequencing
- **Supplemental Table 1.** MIC breakpoints and ECOFF values used to consider isolates as resistant to fluconazole and voriconazole (mg/L)
- **Supplemental Table 2.** Repartition of the rare species (involved in less than 6 episodes) in children in comparison to those species in adult population
- **Supplemental Table 3.** Characteristics of patients with resistant species fungemia
- **Graphical abstract**

Institut Pasteur

Centre National de Référence Mycoses Invasives et Antifongiques
INSTITUT PASTEUR
Pr. Françoise Dromer (directeur)
Pr. Olivier Lortholary et Pr. Stéphane Bretagne (adjoints) – Dr. Alexandre Alanio (collaborateur)
28, rue du Dr. Roux, 75724 PARIS Cedex 15
Téléphone : 01 45 68 83 54 ; Télécopie : 01 45 68 84 20 ; email : cnrma@pasteur.fr

OBSERVATOIRE DES LEVURES

CENTRE PARTICIPANT **PATIENT**

Nom du correspondant :	NOM (3 premières lettres)					Prénom (initiale) :	
Hôpital :	Sexe ☐ **M** ☐ **F**	Date de naissance					

A la date de la fongémie, SERVICE D'HOSPITALISATION ……………………………………………………
Date d'hospitalisation :
Service de soins intensifs : ☐OUI ☐NON

SOUCHE ENVOYEE :

**Date de prélèvement** : ……. /…….. /……..	**Site de prélèvement** : ☐ KT veineux central ☐ Site non précisé ☐ Périphérique
**Date de positivité** : ……. /…….. /……..	
**Identification :**	**Présence d'une endocardite** : ☐OUI ☐NON

Précisions :

PATIENT : FACTEURS FAVORISANTS POTENTIELS

**Présence d'un facteur de risque** :	**Sérologie VIH**	**CD4/mm3** :	% CD4 :
☐OUI ☐NON	☐Positive ☐Négative ☐ non connue	………………	…………………

**Chirurgie récente** (< 1 mois) ☐OUI ☐NON		**Cancer / hémopathie** ☐OUI ☐NON		**Transplantation d'organe** ☐OUI ☐NON	
☐ Œsophage – estomac	☐ Pancréas	☐ Digestif	☐ Leucémie aiguë	☐ Rein	
☐ Foie – voies biliaires	☐ Gynécologie	☐ ORL	☐ Lymphome	☐ Cœur	☐ Poumon
☐ Grêle/colon /rectum	☐ Vasculaire	☐ Génital	☐ Hémoglobinopathie	☐ Foie	☐ grêle
☐ Rein – voies urinaires	☐ Orthopédie	☐ Urinaire	☐ Autre	☐ Autre	
☐ Cardiaque	☐ Info. non disponible	☐ Autre	☐ Info. non disponible	☐ Info. non disponible	
☐ Neurochirurgie		☐ Info. non disponible			

Ancienneté du facteur favorisant principal : ☐ < 1 semaine ☐ 1 – 6 mois ☐ ≤ 1 mois ☐ > 6 mois

Précisions éventuelles

**AUTRES FACTEURS**		**MATERIEL ETRANGER**	
☐ Allogreffe de moelle/CSP	☐ Brûlures	☐ KT veineux central	☐ Drains
☐ Autogreffe de moelle/CSP	☐ Colonisation fongique muqueuse	☐ Chambre implantable	☐ Prothèse articulaire
☐ GVH	☐ Traumatisme abdominal	☐ KT artériel	☐ Dériv. ventriculo-péritonéale
☐ Corticoïdes au long cours	☐ Toxicomanie i.v.	☐ Fistule artério-veineuse	☐ Sonde urinaire
☐ Immunosuppresseurs	☐ Autre facteur	☐ Prothèse vasculaire	☐ Autre
☐ Anti-TNF alpha		☐ Prothèse valvulaire	☐ Information non disponible
☐ Antibiothérapie large spectre		☐ Pacemaker	

TRAITEMENT ANTIFONGIQUE

**Avant le diagnostic de fongémie** (durée > 1 mois)		**Après le diagnostic de fongémie** (durée > 48 h)	
☐ AmphotéricineB déoxycholate	☐ Dérivé lipidique AMB	☐ AmphotéricineB déoxycholate	☐ Dérivé lipidique AMB
☐ 5 fluorocytosine	☐ Fluconazole	☐ 5 fluorocytosine	☐ Fluconazole
☐ Itraconazole	☐ Voriconazole	☐ Itraconazole	☐ Voriconazole
☐ Caspofungine	☐ Posaconazole	☐ Caspofungine	☐ Posaconazole
☐ Micafungine	☐ Anidulafungine	☐ Micafungine	☐ Anidulafungine
☐ Autre …………………	☐ Pas de traitement	☐ Autre …………………	☐ Pas de traitement

DEVENIR 30 JOURS APRES LA FONGEMIE :

☐ Vivant ☐ Décédé **Date du décès** : ……./………/……… ☐ Perdu de vue ☐ Information non disponible

Commentaires : ……………………………………………………………………………………………

Ce document est à l'usage exclusif de l'Institut Pasteur – Reproduction & diffusion interdite

Supplementary material

Species identification

For all isolates, chromogenic medium (BBL Chromagar Candida medium; BD, GmbH) or Niger seed agar for *Cryptococcus* spp were used to checked purity. Phenotypic identification was performed using carbon assimilation profiles (ID32C; bioMérieux, Marcy-l'Etoile, France) before 2014 and matrix-assisted laser desorption ionization–time of flight mass spectrometry (MALDI-TOF MS) (MALDI Biotyper; Bruker Daltonik, GmbH) since 2014. Duplex PCR was performed to differentiate *Candida dubliniensis* and *Candida albicans* (Donnelly SM, Sullivan DJ, Shanley DB, Coleman DC. 1999. Phylogenetic analysis and rapid identification of *Candida dubliniensis* based on analysis of ACT1 intron and exon sequences. *Microbiology* 145:1871–1882.). For all isolates, except for *N. glabratus*, *C. tropicalis*, *P. kudriavzevii*, and *K. marxianus* PCR and sequencing of internal transcribed spacer 1 (ITS1)-5.8S-ITS2 and D1D2 regions were performed, using primers V9D/LS266 (de Hoog GS, van den Ende GAH. 1998. Molecular diagnostics of clinical strains of filamentous basidiomycetes. *Mycoses* 41:183–189; Masclaux F, Gueho E, de Hoog GS, Christen R. 1995. Phylogenetic relationships of human-pathogenic *Cladosporium (Xylohypha*) species inferred from partial LS rRNA sequences. *J Med Vet Mycol* 33:327–338.) and NL1/NL4, respectively (O'Donnell K. 1993. *Fusarium* and its near relatives, p 225–233. *In* Reynolds DR, Taylor JW (ed), *The fungal holomorph: mitotic, meiotic and pleomorphic speciation in fungal systematics.* CAB International, Wallingford, United Kingdom.). In addition, part of the actin gene (for *C. lusitaniae* (Guzman B, Lachance MA, Herrera CM. 2013. Phylogenetic analysis of the angiosperm-floricolous insect-yeast association: have yeast and angiosperm lineages co-diversified? *Mol Phylogenet Evol* 68:161–175.) and *Debaryomyces* species (Martorell P, Fernandez-Espinar MT, Querol A. 2005. Sequence-based identification of species belonging to the genus *Debaryomyces*. *FEMS Yeast Res* 5:1157–1165.)), part of the RPBI gene (for *M. guilliermondii*, *M. caribbica*, and *C. carpophila* (Desnos-Ollivier M, Bretagne S, Boullie A, Gautier C, Dromer F, Lortholary O, French Mycoses Study Group. 2019. Isavuconazole MIC distribution of 29 yeast species responsible for invasive infections (2015–2017). *Clin Microbiol Infect* 25:634.e1–634.e4.))or the IGS1 region (for *Trichosporon* species (Sugita T, Nakajima M, Ikeda R, Matsushima T, Shinoda T. 2002. Sequence analysis of the ribosomal DNA intergenic spacer 1 regions of *Trichosporon* species. *J Clin Microbiol* 40:1826–1830.)) was amplified by PCR and sequenced by Sanger sequencing. Sequences were compared to sequences of the type strain of each species and of the closely related species. The species for *Cr. neoformans* complex isolates was determined by PCR amplification of part of the *GPA1* and *PAK1* genes using a triplex PCR with primers specific for the serotype previously described (Lengeler KB, Cox GM, Heitman J. 2001. Serotype AD strains of Cryptococcus neoformans are diploid or aneuploid and are heterozygous at the mating-type locus. *Infect Immun* 69:115–122).

Antifungal susceptibility

Minimal Inhibitory concentratsion (MIC) were determined for all isolates for fluconazole, posaconazole, voriconazole, micafungin, caspofungin, amphotericinB and flucytosine Alsachim (Shimadzu Group Company, France); according to the EUCAST broth microdilution method (ref 19) in 96-well, flat bottom, clear polystyrene, sterile tissue culture plates (product no. 92096; TPP Techno Plastic Products AG, Switzerland). Quality Control strains (ATCC 22019 and ATCC 6258) were included in each set. Additionally, caspofungin, micafungin and amphotericin B MICs were determined with a modified version of the protocol (briefly, antifungal agents were diluted in AM3 medium instead of RPMI medium, as previously described [8]). MIC50 and MIC90 were respectively calculated for each species as values inhibiting at least 50% and 90% of their isolates.

FKS sequencing

Hot Spot (HS) 1 and 2 regions of the *FKS* gene were sequenced for *C. albicans*, C. *dubliniensis, C. tropicalis,* isolates having AM3-MIC caspofungin ≥ 0.5 mg/L, with primers listed previously described [GSC1f/GSC1r for *C. albicans* and *C. dubliniensis* (Park S, Kelly R, Kahn JN, Robles J, Hsu MJ, Register E, Li W, Vyas V, Fan H, Abruzzo G, Flattery A, Gill C, Chrebet G, Parent SA, Kurtz M, Teppler H, Douglas CM, Perlin DS. 2005. Specific substitutions in the echinocandin target Fks1p account for reduced susceptibility of rare laboratory and clinical *Candida* sp. isolates. *Antimicrob Agents Chemother* 49:3264–3273.); primers CTS1-1f/CTS1-1r for *C. tropicalis* (Desnos-Ollivier M, Bretagne S, Raoux D, Hoinard D, Dromer F, Dannaoui E. 2008. Mutations in the *fks1* gene in *Candida albicans, C. tropicalis*, and *C. krusei* correlate with elevated caspofungin MICs uncovered in AM3 medium using the method of the European Committee on Antibiotic Susceptibility Testing. *Antimicrob Agents Chemother* 52:3092–3098)].Reference strains (*C. albicans* CBS 562, *C. dubliniensis* CBS 7987, *C. tropicalis* ATCC 750,) were used as positive control for PCR amplification and as wild-type reference for *FKS* protein sequences.

Supplemental Table 1. MIC breakpoints and ECOFF values used to consider isolates as resistant to fluconazole and voriconazole (mg/L)

	C. albicans, C. dubliniensis, C. tropicalis, C. parapsilosis	***C. lusitaniae***	***N. glabratus* (syn. *C. glabrata*)**	***P. kudriavzevii* (syn. *C. krusei*)**	***K. marxianus* (syn, *C. kefyr*)**
**fluconazole**	> 4	> 2	> 16	NA	>1 (TECOFF)
**voriconazole**	> 0.25	> 0.03	>1 (ECOFF)	> 1	> 0.03

[#]*values in mg/L*
**C., Candida; N., Nakaseomyces; K., Kluyveromyces; P., Pichia*

Supplemental Table 2. Repartition of the rare species (involved in less than 6 episodes) in children in comparison to those species in adult population

	**Children** n=24 (%)	**Adults** n=415 (%)
Rhodotorula mucilaginosa	4 (16.7)	14 (3.4)
Cryptococcus neoformans complex	2 (8.3)	154 (37.1)
Meyerozyma guilliermondii	2 (8.3)	28 (6.7)
Saccharomyces cerevisiae	2 (8.3)	15 (3.6)
Lodderomyces elongisporus	2 (8.3)	0 (0)
Candida orthopsilosis	1 (4.2)	22 (5.3)
Candida metapsilosis	1 (4.2)	18 (4.3)
Cyberlindnera fabianii	2 (8.3)	2 (0.5)
Meyerozyma caribbica	1 (4.2)	11 (2.6)
Candida haemulonii	1 (4.2)	3 (0.7)
Candida inconspicua	1 (4.2)	13 (3.1)
Candida rugosa	1 (4.2)	3 (0.7)
Trichosporon mucoides	1 (4.2)	0 (0)
Magnusiomyces capitatus	1 (4.2)	15 (3.6)
Magnusiomyces clavatus	1 (4.2)	12 (2.9)
Kodamaea ohmeri	1 (4.2)	5 (1.2)

Supplemental Table 3. Characteristics of patients with resistant species fungemia

	Resistant or non-WT isolates			
	**Caspofungin** (n=11)	**Fluconazole** (n=24)	**Voriconazole** (n=8)	**5-FC** (n=16)
**Species**				
C. albicans	2 (18.2)	1 (4.2)	1 (12.5)	12 (75.0)
C. parapsilosis	0 (0)	2 (8.3)	1 (12.5)	0 (0)
C. tropicalis	1 (9.0)	2 (8.3)	2 (25.0)	3 (18.8)
C. lusitaniae	0 (0)	0 (0)	1 (12.5)	1 (6.2)
N. glabratus (syn. C. glabrata)	0 (0)	6 (25.0)	3 (37.5)	0 (0)
K. marxianus (syn, C. kefyr)	0 (0)	2 (8.3)	0 (0)	0 (0)
P. kudriavzevii (syn. C. krusei)	0 (0)	9 (37.5)	0 (0)	0 (0)
Trichosporon asahii	7 (63.6)	2 (8.3)	0 (0)	0 (0)
C. dubliniensis	1 (9.0)	0 (0)	0 (0)	0 (0)
**Sex Male /Female**	5/6 (45.5)	14/10 (58.3)	5/3 (62.5)	11/5 (68.8)
**Age, mean + SD**	7,42 +-6.02	7,87 +- 5,08	8,64 +-3,91	4,06 +-3,69
**Previous antifungal treatment**	6 (54.5)	9 (37.5)	4 (50.0)	0 (0)
Triazoles	3 (27.2)	3 (12.5)	0 (0)	0 (0)
Echinocandin	5 (45.5) [#]	5 (20.8)	3 (37.5)	0 (0)
Amphotericin B	1 (9.0)	2 (8.3)	1 (12.5)	0 (0)
**First-line of treatment**	11 (100)	23 (95.8)	7 (87.5)	14 (87.5)
Echinocandin	2 (18.2)	15 (62.5)	5 (62.5)	8 (50.0)
Amphotericin B	5 (45.5)	5 (20.8)	3 (37.5)	4 (25.0)
Triazoles	5 (45.5)	5 (20.8)	0 (0)	2 (12.5)
5-FC	1 (9.0)	1 (4.2)	1 (12.5)	0 (0)
**Overall death at 30 days**	4/8(50) [##]	5/24 (20.8)	4/8 (50)	2/15(13.3) [##]

**C., Candida; N., Nakaseomyces; K., Kluyveromyces; P., Pichia; SD, standard deviation; 5-FC, fluocytosine*
[#]C. albicans (n=2), C. tropicalis (n=1), T. asahii (n=2); [##]missing data

Paediatric Yeast Fungemia in France: Twenty Years of Surveillance Reveal Distinct Species Patterns, Resistance Profiles and Outcomes

YEASTS program 2002-2022

24 tertiary-care centers → Yeast → CNRMA

standardized questionnaire

- Confirm species identification
- EUCAST sensitivity profile

→

- Analyse of characteristics of pediatric first episodes of yeast bloodstream infections
- Comparison with adult population

Children's characteristics

346 episodes in children aged 6m to 16 years

♂ 60.1%

16.5% **preexposed to antifungal** (echinocandins ++ after 2012)

Antifugal treatment :
44% echinocandins / 14% L-AMB / 14% triazoles

Species involved (%)

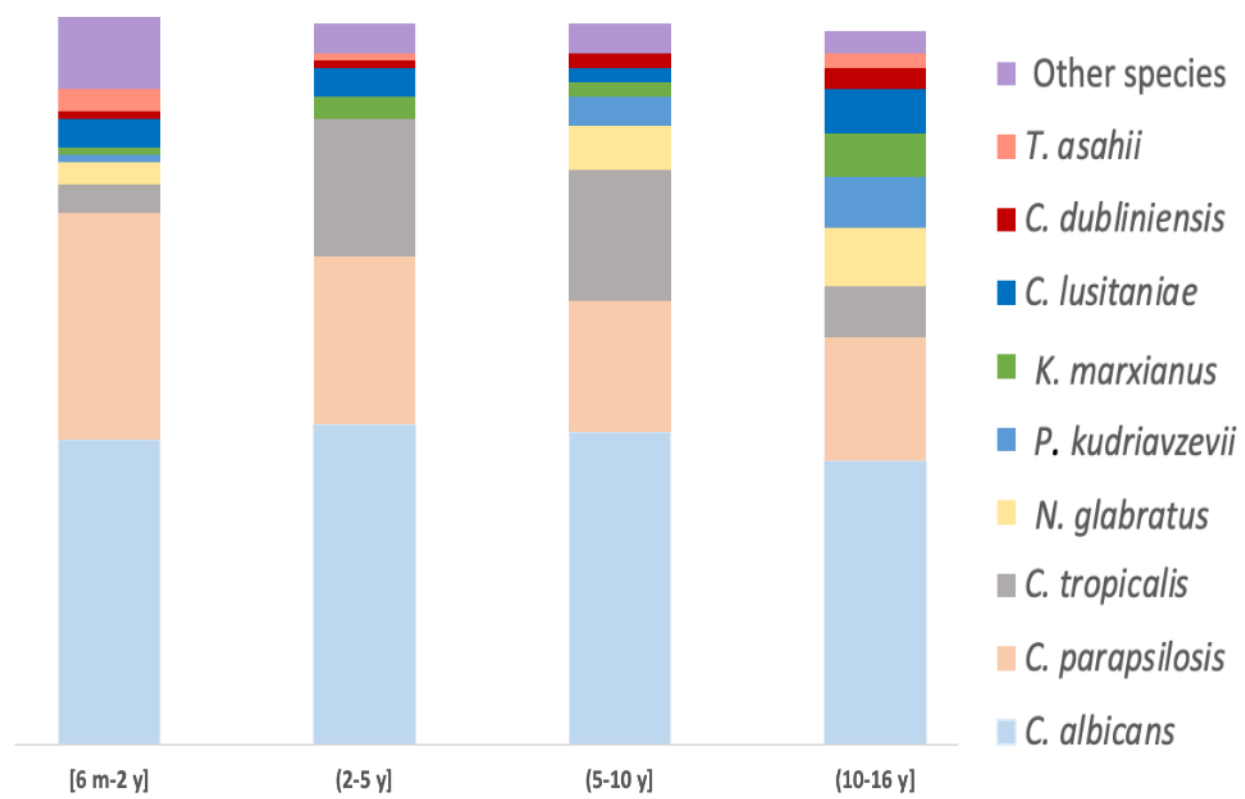

Resistance to caspofungin → **4% of isolates**

Resistance to fluconazole → **8.1% of isolates**

Main risk factor

**Hematological malignancy**	**20.5%**
**Recent surgery**	**19.4%**
**Solid tumor**	**13%**
**HSCT**	**6.7%**
**Solid Organ Transplantation**	**3.5%**

Survival

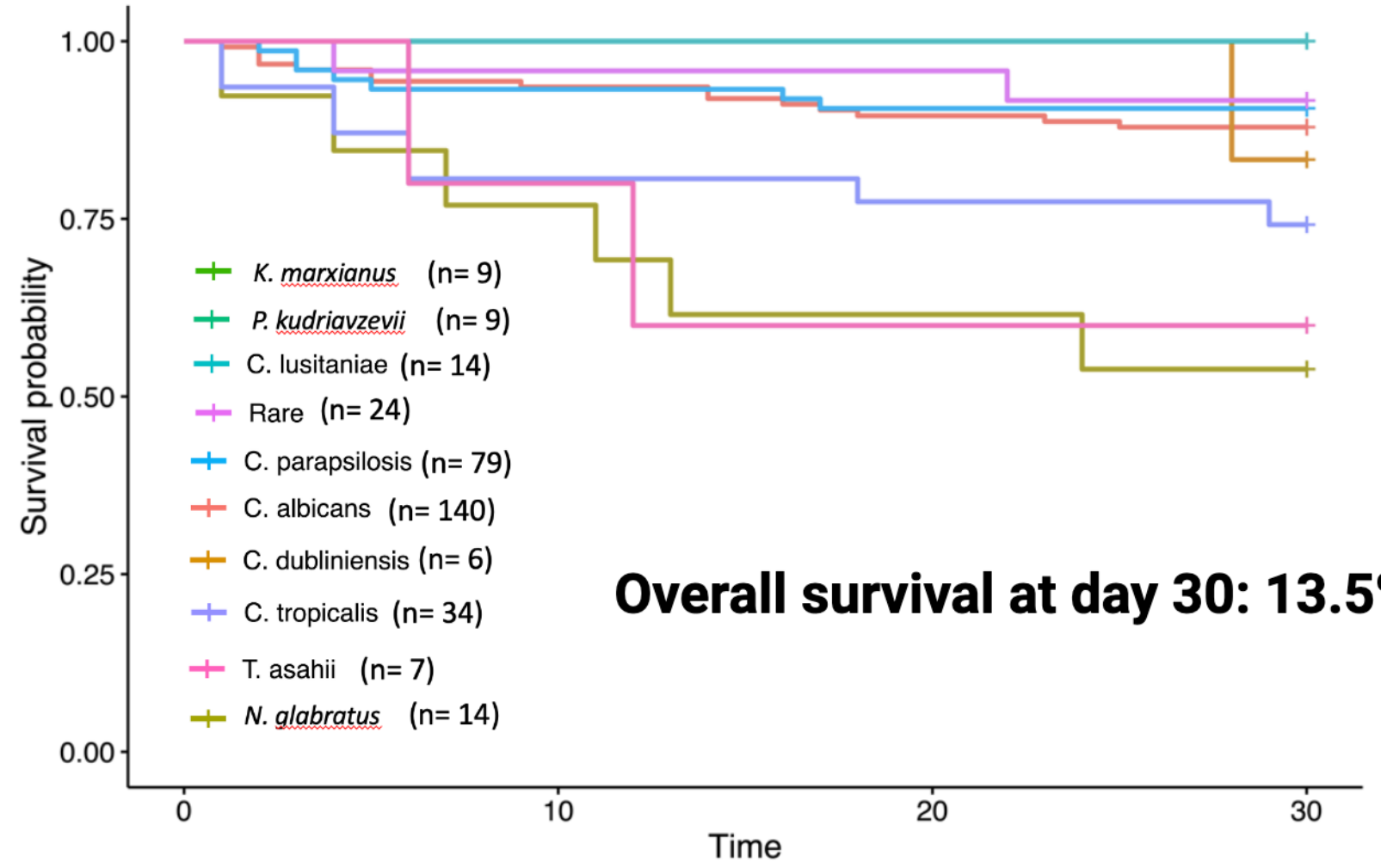

Overall survival at day 30: 13.5%

Comparison with adult population

➕ *C. parapsilosis* and *T. asahii* (p<0.001)

➖ *N. glabratus* (p<0.001)

↑ 16.5% vs 11.1% (p<0.001)

↓ 13.5% vs 40.9% (p<0.001)

➕ L-AMB : 14% vs 5% (p<0.001)
