# Supplementary material for: Pediatric yeast fungaemia species patterns, resistance profiles, and outcomes: a 20-year surveillance study in Paris, France
Source: eClinicalMedicine. 2026 Sep 1;99:104177. doi: 10.1016/j.eclinm.2026.104177 (PMC13571122; doi:10.1016/j.eclinm.2026.104177)
Supplement: Supplementary file 2 [file mmc2.docx]

The French Mycoses Study Group is composed of the following individuals who actively participated in the data collection. They are listed in alphabetical order of a city in France and for each center, in alphabetical order of the last names:

Alexandre Alanio (hôpital Saint Louis, Paris) ; Adela Angoulvant (hôpital du Kremlin Bicêtre, Kremlin-Bicêtre) ; Nawel Ait-Ammar (hôpital Le Raincy-Montfermeil) , Christine Bonnal (hôpital Bichat, Paris), Sophie Brun (hôpital Bichat, Paris), Naima Dahane (hôpital Cochin, Paris), Blandine Denis (hôpital Saint Louis, Paris), Samia Hamane (hôpital Saint Louis, Paris), Najiby Kassis-Chikhany (hôpital Européen Georges Pompidou, Paris) , Maïté Micaelo (Centre Hospitalier, Versailles), André Paugam (hôpital Cochin, Paris), Anne-Laure Roux (hôpital Ambroise Paré, Boulogne).
